# Fluorometric Quantification of Total Cell-Free DNA as a Prognostic Biomarker in Non-Small-Cell Lung Cancer Patients Treated with Immune Checkpoint Blockade

**DOI:** 10.3390/cancers15133357

**Published:** 2023-06-26

**Authors:** Javier Oliver, Juan Luis Onieva, María Garrido-Barros, Manuel Cobo-Dols, Beatriz Martínez-Gálvez, Ana Isabel García-Pelícano, Jaime Dubbelman, José Carlos Benítez, Juan Zafra Martín, Alejandra Cantero, Elisabeth Pérez-Ruiz, Antonio Rueda-Domínguez, Isabel Barragán

**Affiliations:** 1 Medical Oncology Service (Group of Translational Research in Cancer Immunotherapy), Regional and Virgen de la Victoria University Hospitals, Institute of Biomedical Research in Malaga and BIONAND Nanomedicine Platform (IBIMA BIONAND Platform), C/Marqués de Beccaría n°3, 29010 Málaga, Spain; javier.oliver@ibima.eu (J.O.);; 2 Faculty of Medicine, Campus de Teatinos s/n, Universidad de Málaga, 29071 Málaga, Spain; 3 Department of Radiation Oncology, Virgen de la Victoria University Hospital, 29010 Málaga, Spain; 4 Group of Pharmacoepigenetics, Department of Physiology and Pharmacology, Karolinska Institutet, 171 77 Stockholm, Sweden

**Keywords:** cfDNA, immunotherapy, NSCLC, prognosis

## Abstract

**Simple Summary:**

The current study investigated the potential use of fluorometric cfDNA quantification as a prognostic biomarker in advanced non-small cell lung cancer (NSCLC) patients treated with an Immune Checkpoint Blockade (ICB). Results showed that a cfDNA cut-off of 0.55 ng/µL before the ICB determines the overall survival of patients with a log rank *p*-value of 3.3 × 10^−4^. High cfDNA concentrations identify patients with advanced NSCLC who do not benefit from ICB. The determination of cfDNA is a simple test that could select a group of patients in whom new therapeutic strategies are needed.

**Abstract:**

The present study aimed to investigate the potential of basal cell-free fluorometric DNA (cfDNA) quantification as a prognostic biomarker in advanced non-small cell lung cancer (NSCLC) patients treated with an Immune Checkpoint Blockade (ICB). A discovery and validation cohort of 61 and 31 advanced lung cancer patients treated with ICB were included in this study. Quantification of cfDNA concentration was performed before the start of the treatment and patients were followed up for a median of 34 (30–40) months. The prognostic predicted value of cfDNA was evaluated based on ROC, and Cox regression was conducted via univariate and multivariate analyses to estimate the hazard ratio. We observed that a cfDNA cut-off of 0.55 ng/µL before the ICB determines the overall survival of patients with a log rank *p*-value of 3.3 × 10^−4^. That represents median survivals of 3.8 vs. 17.5 months. Similar results were obtained in the validation cohort being the log rank *p*-value 3.8 × 10^−2^ with median survivals of 5.9 vs. 24.3. The univariate and multivariate analysis revealed that the cut-off of 0.55 ng/µL before ICB treatment was an independent predictive factor and was significantly associated with a better survival outcome. High cfDNA concentrations identify patients with advanced NSCLC who do not benefit from the ICB. The determination of cfDNA is a simple test that could select a group of patients in whom new therapeutic strategies are needed.

## 1. Introduction

Lung cancer is one of the deadliest solid cancers worldwide [1,2]. Metastatic disease is present in about 60% of lung cancer cases, and the 5-year survival rate is still under 10% [3]. The irruption of immunotherapy based on the Immune Checkpoint Blockade (ICB) with anti-PD-(L)1 monoclonal antibodies was considered a revolution in the treatment of advanced lung cancer, particularly Non-Small Cell Lung Cancer (NSCLC). Currently, the ICB is used in combination with platinum doublet chemotherapy or in monotherapy, mainly depending on the PD-L1 levels [4].

Despite the potential of ICB approaches to treat malignancies of bad prognoses, its efficient use is limited because the mechanisms leading to resistance are not defined, hence the lack of definitive predictive biomarkers. Approximately 20% of unselected patients have long-term benefits from the ICB [5]. Indeed, a relevant number of patients that do respond end up developing secondary resistances over time, requiring a new line of systemic therapy which is usually less effective and has a worse toxicity profile [6]. In addition to the subsequent impact on patient survival, the identification of resistance biomarkers for patient selection is critical given the escalating costs of this type of treatment, which currently ranges from 50,000 to 100,000$/quality-adjusted life year [7].

The only FDA-approved biomarker specific of the ICB specific for NSCLC, PD-L1, is based on immunohistochemistry [8]. Its utility for selecting patients for therapy is hampered by the unclear definition of PD-L1 positivity and at least some potential for therapeutic response regardless of tumor PD-L1 status [9,10]. Indeed, even though there are four IHC assays approved by the FDA, protein PD-L1 expression fails to accurately predict the response to the ICB in some cases [11].

Other approved biomarkers useful for ICB-related therapeutic decisions are the tumor mutational burden (TMB) [5], detected by Next Generation Sequencing (NGS) panels such as mSK-ImPACT^®^ and FoundationOne CDx^®^, and the agnostic biomarker based on deficiencies of the Mismatch Repair system detected with PCR or Immunohistochemistry panels such as VENTANA MMR RxDx^®^ [12]. However, the methods that depend on invasive solid biopsies and rely on complex technologies are far from providing accurate and precise biomarkers that can be implemented in the Health Systems to identify patients with a durable clinical response before or early during treatment. Therefore, developing efficient and simpler treatment prognosis indicators is an urgent need [13].

The cell-free DNA (cfDNA) pool is fed mainly by the normal blood cells, primary tumor, metastases, and circulating tumor cells. The release of cfDNA varies among cancer types and its association with a high number of metastases, tumor burden, and advanced stage is well established [14]. However, its relation to clinical benefits of therapy is not fully explored. Among the scarce reports, a ≥20% decrease after 6 weeks of chemotherapy relates to better outcomes in NSCLC patients (16.5 months of follow up) [15].

Compared with common methods used for profiling prognostic biomarkers in liquid biopsy, such as droplet digital PCR (ddPCR) or NGS, the fluorometric determination of the cfDNA concentration is a simple, easy, and scalable method to implement. Since the ICB mechanism of action stands on the interaction between immune and tumor cells, we hypothesized that the overall amount of cfDNA in cancer patients would exhibit a stronger correlation with ICB effect. Therefore, in this prospective study, our aim was to evaluate the utility of pre-treatment cfDNA concentration to predict the prognosis of NSCLC patients that are to receive anti-PD-(L)1 antibodies.

## 2. Materials and Methods

### 2.1. Sample Processing and DNA Isolation and Quantification

The discovery (n = 61) and validation (n = 31) cohorts included advanced NSCLC patients that started treatment with the ICB at Hospitals Regional Universitario and Virgen de la Victoria of Málaga from 2019 to 2022. They were clinically and analytically homogeneous, showing no statistical differences for the clinical variables included in the study (Table 1). Both cohorts were collected independently and included the basal pre-treatment liquid biopsy sample. During the study, a clinical and imaging evaluation were performed every 3 months and patients were followed for a median of 34 months (range 30–40). Written informed consent was obtained from all individuals, and the study was approved by the local ethics committee of Malaga (26 October 2017). The study was conducted following REMARK guidelines [16] and the requirements proposed by Simon et al. [17].

Just before the start of the treatment with the ICB, blood samples were collected in CellSave tubes (Menarini Silicon Biosystem Inc., Castel Maggiore, Italy). Then, samples were centrifuged at 1600 rpm for 10 min. The remaining plasma was centrifuged at 4750 rpm for 10 min. Plasma samples were stored at −80 °C in 3 mL cryovials. cfDNA was isolated from plasma with the QIAamp Circulating Nucleic Acid kit (Qiagen, Germantown, MD, USA, 55114) according to the manufacturer’s protocol. The quantity of cfDNA was measured by 1X Qubit High Sensitivity (Thermo Fisher Scientific, Waltham, MA, USA). Fragment size, quality, and quantity of random samples were evaluated by the Bioanalyzer 2100 (Agilent Technologies, Santa Clara, CA, USA).

### 2.2. Statistical Methods

To assess the study variables, a descriptive analysis was conducted to calculate the median values and range (maximum and minimum values). For categorical variables, the results are reported in terms of absolute and relative frequencies. To ensure similar cohort distributions, the clinical variables were compared between the discovery and validation cohorts using the Fisher’s exact or Chi-squared tests for qualitative variables or the Wilcoxon Test for continuous variables. Sample size estimation for the discovery cohort was of 66 patients with a confidence level of 90% and confidence interval of 10%. For the survival analysis, we used a maximally selected log-rank statistic [18] to dichotomize continuous variables. This allows us to assess a threshold value that categorizes observations into two groups based on an ordinal predictor variable. In this case, we dichotomized cfDNA concentration in a 0.55 ng/µL cut-off. Survival probabilities were estimated with the Kaplan–Meier method and survival curves were compared using the log-rank test. Univariate and multivariate Cox regression models were executed to estimate the hazard ratios. We used the Area Under the Curve (AUC) for a right-censored time-to-event estimator to evaluate the performance of the different Cox regression models. Statistical analyses were performed using the R software version 4.0.2.

## 3. Results

### 3.1. Cohorts Characteristics and Sample Collection

The most frequent histology was lung adenocarcinoma (LUAD) (64%), with one third of patients affected by squamous carcinoma (SCC). Male patients were the majority in both cohorts, and ages ranged from 43 to 85 years old. Most of the patients also presented an acceptable performance (ECOG score < 2). Lymph node metastases were the most frequent, followed closely by lung metastases. The treatment schema of approximately 60% of the patients included a combination with chemotherapy, while monotherapy ICB was administered to around one third of the patients (Table 1).

### 3.2. CfDNA Correlates with Response to ICB in NSCLC Patients

**Figure 1:**
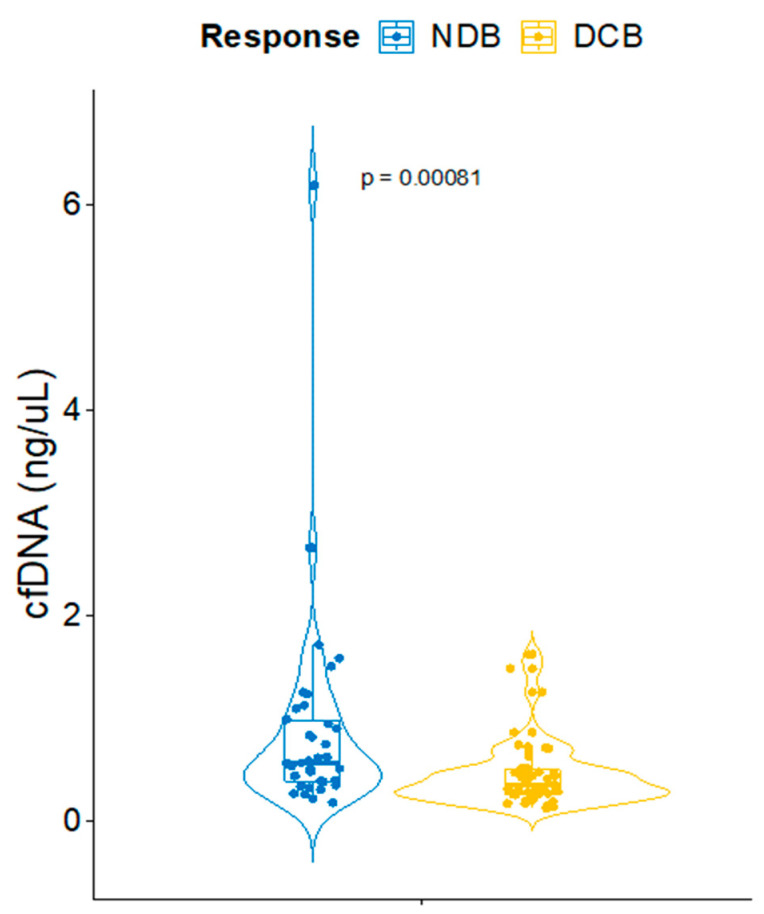
Fluorometric cfDNA concentration values in Non-Durable Benefit (NDB) and Durable Clinical Benefit (DCB) patients.

The high innate resistance rates associated with the ICB in NSCLC makes it urgent to anticipate the response using a biomarker that can be assessed at the moment of the therapeutic decision. Pursuing this goal, we evaluated the basal concentration of cfDNA as a predictor of response to the ICB. The patients of the combined cohort were categorized according to the Durable Clinical Benefit (DCB). This was defined as achieving a partial/complete response or stable disease for more than 6 months. Interestingly, non-DCB (NDCB) patients yielded a 38.9% higher level of basal cfDNA concentration compared with those patients that ultimately achieved the DCB (median cfDNA concentration of 0.559 vs. 0.3415, Wilcoxon Test *p*-value 0.00081) (Figure 1).

### 3.3. Prognostic Analysis and Models

**Figure 2:**
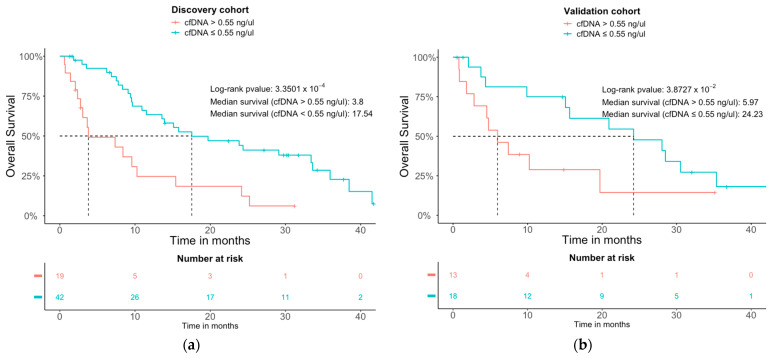
Kaplan-Meier overall survival (OS) curves for patients with high versus low cfDNA in the discovery (**a**) and the validation cohorts (**b**) High and low grouping refers to the basal cfDNA concentration threshold of 0.55 ng/µL.

We also explored the prognosis potential of the cfDNA concentration on the therapeutic response to anti-PD-(L)1. With the intention of establishing a quantitative cut-off value useful to stratify the patients according to the survival risk, we conducted survival studies in the discovery cohort and tested the obtained cut-off values in the validation cohort. Patients with cfDNA concentrations over 0.55 ng/µL showed worse OS in both cohorts (log-rank *p*-value: 3.35 × 10^−3^ and log-rank *p*-value: 3.87 × 10^−2^) compared with the patients with a cfDNA concentration lower than 0.55 ng/µL (Figure 2). The median survival for the discovery and validation cohorts for patients with a cfDNA concentration lower than 0.55 ng/µL was of 17.5 and 24.23 months, respectively, compared to 3.8 and 5.9 months for the patients with a higher cfDNA concentration.

**Figure 3:**
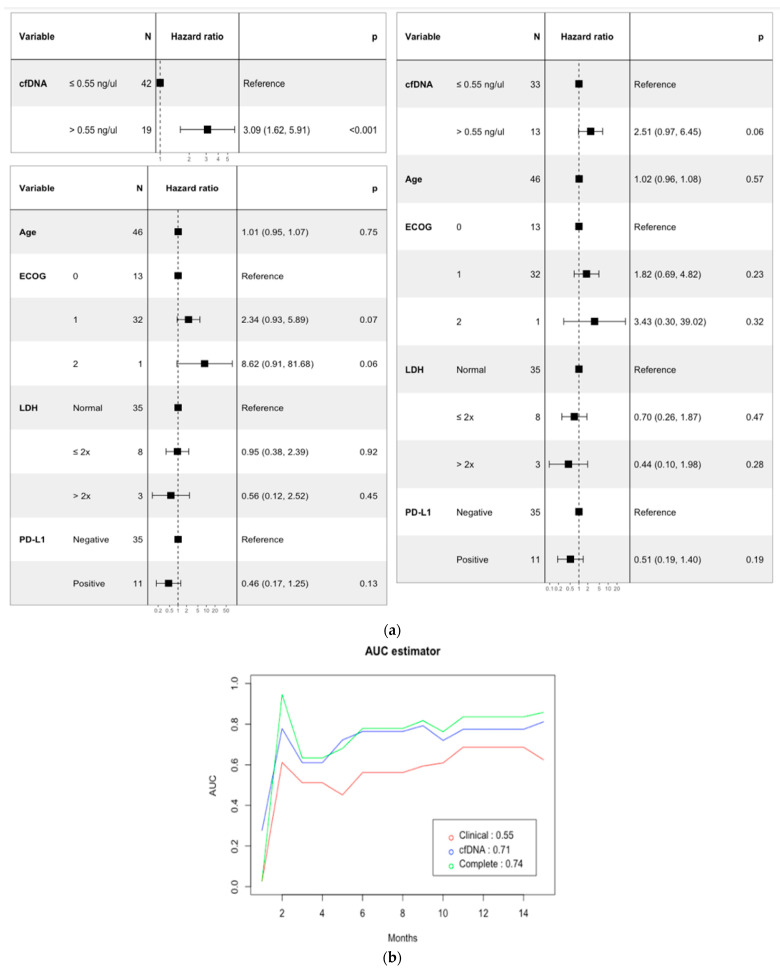
Prognostic models using cfDNA and combinations of cfDNA and clinico-pathological features. (**a**) Univariate and multivariate analyses of prognostic factors of OS. (**b**) Comparison of the prognostic potential of the cfDNA prognostic models using the AUC estimator.

In order to build the prediction models, cfDNA concentration, age at treatment, *Eastern Cooperative Oncology Group* score (ECOG score), Lactate dehydrogenase (LDH), and PDL1 status were selected for univariate and multivariate Cox regression analyses (Figure 3a). cfDNA concentrations over 0.55 ng/µL were also associated with worse outcomes and were independent predictors for OS with a hazard ratio of 3.09 CI (1.6–5.9) and *p* value < 0.001. Finally, the potential prognosis value of cfDNA concentration was compared with the established model based on the mentioned variables using the AUC estimator proposed by Uno et al. [19]. The summary AUC output of our cfDNA model is 0.71, whereas the clinical model has an AUC of 0.55. Moreover, the combination of the cfDNA concentration and age at treatment, and ECOG, LDH, and PDL1 status outperforms the individual model by reaching a summary AUC of 0.74 (Figure 3b). At months 2 and 14, the multivariable model reaches the maximum AUCs of 0.94 and 0.83, respectively.

## 4. Discussion

The present study evidences a significant association between total plasma cfDNA and ICB response and prognosis in NSCLC patients. The basal concentration of cfDNA is not only disbalanced between DCB and NDCB patients but can be converted into an easily implementable test where cfDNA values below 0.55 ng/µL predict a high probability of long-term survival. The large follow up of this study is particularly important considering that we are assessing the clinical benefit of immunotherapy in patients with an expected bad prognosis as advanced NSCLC patients. Indeed, median survival in patients stratified according to the 0.55 ng/µL threshold is of 17.5 and 24.23 months, depending on the cohort in patients with levels lower than the cut-off value, but is strikingly shorter in those patients with more than 0.55 ng/µL of basal cfDNA: 3.8 and 5.9 months for the discovery and validation cohorts, respectively.

The association of circulating tumor DNA (ctDNA) with prognosis has been deeply studied. Indeed, certain somatic tumor mutations such as T790M in *EGFR* detected in blood have clinical validity for the EGFR tyrosine kinase inhibitor selection in NSCLC [20]. Moreover, in cancer patients of several types treated with the ICB, both pre-treatment ctDNA and dynamic changes in response to treatment are prognostic biomarkers [21]. However, the utility of global cfDNA concentration in cancer therapy response is far less explored because tumor specificity is presumed to be low.

Of the few available studies, the potential of cfDNA quantification as a prognostic biomarker has been highlighted in metastatic melanoma, where it surrogated the tumor burden [22], or in prostate cancer, indicating a particular capacity for identifying metastatic castration-resistant prostate cancer. However, differences in cfDNA amounts failed to distinguish between healthy individuals and patients with localized prostate cancer [23]. In the specific context of ICB treatment, one study in 85 patients with hepatocellular carcinoma (HCC) treated with the combination of PD-L1 and anti-vascular endothelial growth factor (VEGF) inhibitors established a correlation of plasma cfDNA levels with response and progression-free and overall survival [24]. The reports related to tumors treated with ICB and in particular, the ones addressed in our study, NSCLC, use the quantification of h*TERT* in cfDNA [25,26].

The cfDNA pool is mainly contributed by white blood cells, and to a minor extent by solid tissues, through diverse cell death mechanisms including necrosis, secretion, and apoptosis [27] and more recently, via NETosis [28]. In cancer patients, the cfDNA composition is skewed towards DNA from tumor cells, from the tumor microenvironment cells, and from cells acting in the antitumor response [27]. Interestingly, cfDNA can also be associated to exosomes and the fraction of both external and internal exosome DNA is enriched in tumor cells [29]. This is coherent with the fact that the range of plasma cfDNA in cancer patients tends to be considerably higher, up to 1 ng/µL, than in healthy subjects, where it varies between 0 to 0.01 ng/µL [30,31]. Regarding this tumor contribution to the cfDNA pool, we can speculate that, similarly to the associations observed for ctDNA, basal cfDNA is not only informative of the tumor burden but reflects complex processes of the biology of the tumor such as active metabolism or aggressiveness that can influence the therapeutic response in the metastatic NSCLC scenario [32].

Apart from the contribution of the cfDNA of tumor origin to the association of global cfDNA with response and prognosis, immune cells’ cfDNA may be relevant for obtaining such particularly high correlations to clinical outcome in the context of response to immunotherapy. High cfDNA is an established marker of inflammation and tissue damage, and proxy of sepsis, autoimmune diseases, and cancer [33]. Neutrophils are the most abundant cell type in human peripheral blood and are crucial for triggering acute inflammatory responses and regulating the innate and adaptive immunity of chronic inflammation [34]. Indeed, a significant fraction of the cfDNA pool includes the neutrophils’ extruded nuclear DNA that conforms the Neutrophil Extracellular Traps (NETs), useful to entrap and kill prokaryotic microorganisms [35]. Interestingly, the presence of NETs is negatively correlated to the abundance of CD8+ T lymphocytes, which is indicative of a tolerogenic immune phenotype [36]. Indeed, intratumor NETs have been reported to protect malignant cells via the adhesive mechanism that is designed to fight microbial pathogens. This mechanism is also associated with tumor metastasis. In this context, the NETosis blockade sensitizes tumors to the ICB combination of PD-1 and CTLA-4 inhibitors and increases the cytotoxic immunity against subcutaneous tumors and metastases [37].

Interestingly, this immunological contribution in the predictive potential of cfDNA in the ICB regimens is coherent with the higher informativity of cfDNA versus ctDNA detection in the study that evaluated the ability of these molecules to predict response and prognosis in HCC patients treated with PD-L1 + VEGF inhibitors. Only specific *TERT* mutations were associated with OS, and not ctDNA presence/absence [24].

In addition to the identification and validation of a non-arbitrary threshold that predicts long-term clinical benefits to the ICB in NSCLC, we established prognosis prediction models based on the univariate and multivariate analyses estimating the quality of the prediction with the AUC estimator. Remarkably, the cfDNA cut-off categorization reaches a clinically useful prediction potential, exceeding in great extent the AUC provided by a multivariant model with age, ECOG, LDH, and PD-L1 expression. Moreover, the AUC of this model based on clinic-pathological variables improves importantly (from 0.55 to 0.74) if cfDNA is included in the algorithm. This is particularly relevant given that a simple and replicable fluorometric quantification would constitute an agnostic biomarker with which we could dispense with personalized tracked tumor markers and increase the negative predictive value [38]. In addition, such a cost-effective and affordable approach confronts disparity and is feasible to validate retrospectively and prospectively in clinical trials.

## 5. Conclusions

In summary, we are the first to report a specific basal cfDNA concentration cut-off to stratify the patients that are to receive the ICB according to long term outcomes. Therefore, it constitutes a non-invasive and reliable tool useful for therapy decision making and for limiting toxicity. The addition of the basal cfDNA concentration increases the sensitivity and specificity of the best clinical prognosis prediction model (age, ECOG, LDH, and PD-L1 expression). We anticipate a high utility potential of this novel prognosis biomarker in the clinical management of NSCLC with ICB.

## Figures and Tables

**Table 1:** Patient characteristics and homogeneity: discovery, validation, and combined cohorts.

Variable	Group	Discovery Cohort	Validation Cohort	Combined Cohort	*p*-Value Discovery vs. Validation	Pct NA—Complete	Pct NA—Discovery	Pct NA—Validation
**Sex**					0.45	0 (0%)	0 (0%)	0 (0%)
	Female	12 (19.67%)	9 (29.03%)	21 (22.83%)				
	Male	49 (80.33%)	22 (70.97%)	71 (77.17%)				
**Age IT Start**		64.56 [43.38–84.9]	66.38 [53.29–79.12]	65.97 [43.38–84.9]	0.75	0 (0%)	0 (0%)	0 (0%)
**Histology**					0.79	1 (1.09%)	1 (1.64%)	0 (0%)
	LUAD	39 (65.00%)	20 (64.52%)	59 (64.83%)				
	Others	2 (3.33%)	0 (0%)	2 (2.20%)				
	SCC	19 (31.67%)	11 (35.48%)	30 (32.97%)				
**Immunotherapy type**					0.30	0 (0%)	0 (0%)	0 (0%)
	ICB-mono	23 (37.7%)	11 (35.48%)	34 (36.96%)				
	ICB + chemo	8 (13.11%)	8 (25.81%)	16 (17.39%)				
	ICB + chemo + Others	30 (49.18%)	12 (38.71%)	42 (45.65%)				
**Brain metastasis**					0.32	5 (5.43%)	3 (4.92%)	2 (6.45%)
	No	48 (78.69%)	27 (87.1%)	75 (81.52%)				
	Yes	10 (16.39%)	2 (6.45%)	12 (13.04%)				
**Lymph node metastasis**					1.00	5 (5.43%)	3 (4.92%)	2 (6.45%)
	No	25 (40.98%)	13 (41.94%)	38 (41.3%)				
	Yes	33 (54.1%)	16 (51.61%)	49 (53.26%)				
**Liver metastasis**					1.00	5 (5.43%)	3 (4.92%)	2 (6.45%)
	No	51 (83.61%)	25 (80.65%)	76 (82.61%)				
	Yes	7 (11.48%)	4 (12.9%)	11 (11.96%)				
**Lung metastasis**					0.82	5 (5.43%)	3 (4.92%)	2 (6.45%)
	No	33 (54.1%)	18 (58.06%)	51 (55.43%)				
	Yes	25 (40.98%)	11 (35.48%)	36 (39.13%)				
**Toxicity to IT (all grades)**					0.97	7 (7.61%)	6 (9.84%)	1 (3.23%)
	No	24 (39.34%)	14 (45.16%)	38 (41.3%)				
	Yes	31 (50.82%)	16 (51.61%)	47 (51.09%)				
**Maximum toxicity grade**					0.18	14 (15.22%)	10 (16.39%)	4 (12.9%)
	0	17 (27.87%)	10 (32.26%)	27 (29.35%)				
	1	23 (37.7%)	6 (19.35%)	29 (31.52%)				
	2	4 (6.56%)	6 (19.35%)	10 (10.87%)				
	3	5 (8.2%)	4 (12.91%)	9 (9.80%)				
	4	1 (1.64%)	1 (3.23%)	2 (2.17%)				
	5	1 (1.64%)	0 (0%)	1 (1.09%)				
**PDL1**						7 (7.61%)	4 (6.56%)	3 (9.68%)
	<50%	44 (72.13%)	23 (74.19%)	67 (72.83%)				
	>50%	13 (21.31%)	5 (16.13%)	18 (19.57%)				
**Previous treatment**					0.73	0 (0%)	0 (0%)	0 (0%)
	No	43 (70.49%)	20 (64.52%)	63 (68.48%)				
	Yes	18 (29.51%)	11 (35.48%)	29 (31.52%)				
**Progression**					1.00	0 (0%)	0 (0%)	0 (0%)
	No	18 (29.51%)	9 (29.03%)	27 (29.35%)				
	Yes	43 (70.49%)	22 (70.97%)	65 (70.65%)				
**State last evaluation**					1.00	0 (0%)	0 (0%)	0 (0%)
	Dead	44 (72.13%)	22 (70.97%)	66 (71.74%)				
	Alive	17 (27.87%)	9 (29.03%)	26 (28.26%)				
**T1 ECOG**					0.79	4 (4.35%)	1 (1.64%)	3 (9.68%)
	0	15 (24.59%)	6 (19.35%)	21 (22.83%)				
	1	43 (70.49%)	22 (70.97%)	65 (70.65%)				
	2	2 (3.28%)	0 (0%)	2 (2.17%)				
**T1 LDH**					0.89	22 (23.91%)	13 (21.31%)	9 (29.03%)
	<=2x	8 (13.11%)	5 (16.13%)	13 (14.13%)				
	>2x	3 (4.92%)	1 (3.23%)	4 (4.35%)				
	Normal	37 (60.66%)	16 (51.61%)	53 (57.61%)				

## Data Availability

Data available upon request and code available on http://github.com/ImmunoOncology.
